# Proportions of the aesthetic African-Caribbean face: idealized ratios, comparison with the golden proportion and perceptions of attractiveness

**DOI:** 10.1186/s40902-018-0161-5

**Published:** 2018-09-05

**Authors:** Angelos Mantelakis, Michalis Iosifidis, Zaid B. Al-Bitar, Vyron Antoniadis, David Wertheim, Umberto Garagiola, Farhad B. Naini

**Affiliations:** 1 London and South East Foundation School, London, UK; 2 St George’s University of London, London, UK; 3 Department of Orthodontics and Pediatric Dentistry, School of Dentistry, University of Jordan, Amman, Jordan; 4 Bloomberg LP, London, UK; 5 Faculty of Science, Engineering and Computing, Kingston University, London, UK; 6 Maxillofacial and Odontostomatology Unit, School of Dentistry, University of Milan, Milan, Italy; 7 Kingston and St George’s Hospitals and St George’s Medical School, Blackshaw Road, London, SW17 0QT UK

**Keywords:** Facial, Aesthetic, Beauty, Proportions, Golden proportions, Perception

## Abstract

**Background:**

In the absence of clear guidelines for facial aesthetic surgery, most surgeons rely on expert intuitive judgement when planning aesthetic and reconstructive surgery. One of the most famous theories regarding “ideal” facial proportions is that of the golden proportion. However, there are conflicting opinions as to whether it can be used to assess facial attractiveness. The aim of this investigation was to assess facial ratios of professional black models and to compare the ratios with the golden proportion.

**Methods:**

Forty photographs of male and female professional black models were collected. Observers were asked to assign a score from 1 to 10 (1 = not very attractive, 10 = very attractive). A total of 287 responses were analysed for grading behaviour according to various demographic factors by two groups of observers. The best graded photographs were compared with the least well-graded photographs to identify any differences in their facial ratios. The models’ facial ratios were calculated and compared with the golden proportion.

**Results:**

Differences in grading behaviour were observed amongst the two assessment groups. Only one out of the 12 facial ratios was not significantly different from the golden proportion.

**Conclusions:**

Only one facial ratio was observed to be similar to the golden proportion in professional model facial photographs. No correlation was found between facial ratios in professional black models with the golden proportion. It is proposed that an individualistic treatment for each ratio is a rather better method to guide future practice.

## Background

For many years, there has been an interest in elucidating whether beauty is an objective, measurable concept, or indeed as many support, whether it lies subjectively in the “eye of the beholder”. Beauty very much affects one’s life, both in personal relationships, where Berscheid et al. [1] advocated that humans attribute positive qualities to attractive faces and negative ones to unattractive people, but also in their professional lives, where less attractive adults are perceived as having fewer qualifications and potential for career success. It is therefore important and relevant to investigate if facial aesthetic appearance is possibly modifiable with scientific and measurable practices in modern surgery, rather than being an arbitrary concept and matter of personal preference.

One of the most famous concepts regarding facial beauty is that of the golden proportion. This geometrical proportion is identified when “a straight line AB is divided at point C in such a way that AB/AC = AC/CB” [2]. It has been hypothesized that the Greek sculptor Phidias used it for the design of the Parthenon in Athens for dedication to the Greek goddess Athena [3], though there is no firm evidence to support this claim [2]. Later in the twentieth century, the mathematician Mark Barr attributed the term “Phi” for this golden proportion [3]. It has also been claimed that Renaissance artists used this proportion in their paintings and sculptures, most famously Leonardo da Vinci, in his painting “The Last Supper” [3], though, again, there is no firm evidence to support this claim, and it does not appear in any of Leonardo’s own notebooks [2]. It has long been questioned, amongst artists and clinicians, whether the golden proportion may correlate with facial beauty.

During the twentieth century, there have been many attempts to examine any relationship between the golden proportion and perceived facial attractiveness. Ricketts [4], in 1982, through the use of frontal and lateral cephalometric radiographs, devised 12 facial ratios, which complied with the golden proportion in faces that he then considered “ideal”. Since then, other published papers have agreed with this result amongst various populations [5, 6], and it is now a popular belief that it can be applied in the facial aesthetics industry. This has also led to the creation of many beauty applications, such as overlaying “masks” over facial photographs to assess the subjects’ degree of facial beauty in terms of its approximation to the mask, all based on the golden proportion [7].

However, there is also conflicting evidence regarding this correlation. The faces of professional models have not always been found to fit the golden proportion [8], and for patients undergoing orthognathic surgery, whilst most subjects were perceived as more attractive after the operation, the proportions were as likely to move towards or away from golden proportions [9]. Furthermore, studies assessing the prevalence of the golden proportion in the general population rather than just attractive faces [10] found that whole populations may indeed exhibit some facial ratios that are similar to golden proportions; therefore, this proportion may indeed be a facial ratio that many faces exhibit rather than a specific measurement that correlates with beauty.

The aim of this investigation was to assess whether a relationship between the golden proportion and perceived attractiveness exists in 2D images of professional black models. Furthermore, the study aimed to investigate proportional ranges of facial ratios in professional black models, which may provide further insights for planning facial aesthetic and reconstructive surgery.

## Methods

### Subjects and sample selection

The sample photographs used were acquired from modelling agencies on social media platforms and used strictly for research purposes [11]. The sample taken was randomized according to the order found, rather than being selected. For this investigation, 40 photographs, 20 males and 20 females, were selected. These were all adult professional black models and the selection process was based on the following criteria:

- Inclusion criteria: The absence of noticeable asymmetry or craniofacial anomalies. Furthermore, the photographs had to be full-face against a white background, with subjects facing forwards with neutral facial expression.
- Exclusion criteria: Any visible scar from trauma or previous facial surgery, apparent loss of tooth structure, and presence of any inanimate objects (piercings, glasses, headbands, etc.) that could cover facial areas.

After the photographs were acquired, they were uploaded into an online survey on SurveyMonkey® and resized to be seen clearly by the participants. As only proportional facial ratios were calculated, and not exact linear distances, photographs did not have to be of a standardized distance from the camera during sample selection. The online survey that participants received included the aims of the study and was voluntary; anonymous and only demographic data (age, gender, ethnicity and profession) were gathered. The survey was then forwarded to university students and orthodontists. Participants were asked to act as an evaluation jury, through appointing an aesthetic evaluation of the face ranging from 0 to 10 (0 = not attractive, 10 = very attractive). A total of 287 responses were gathered, and the photographs of the models who received the highest frequency of high grades (8, 9 or 10) were considered “best graded models” and the ones with the lowest frequency “not well-graded models”.

### Anthropometric facial measurements

**Fig. 1:**
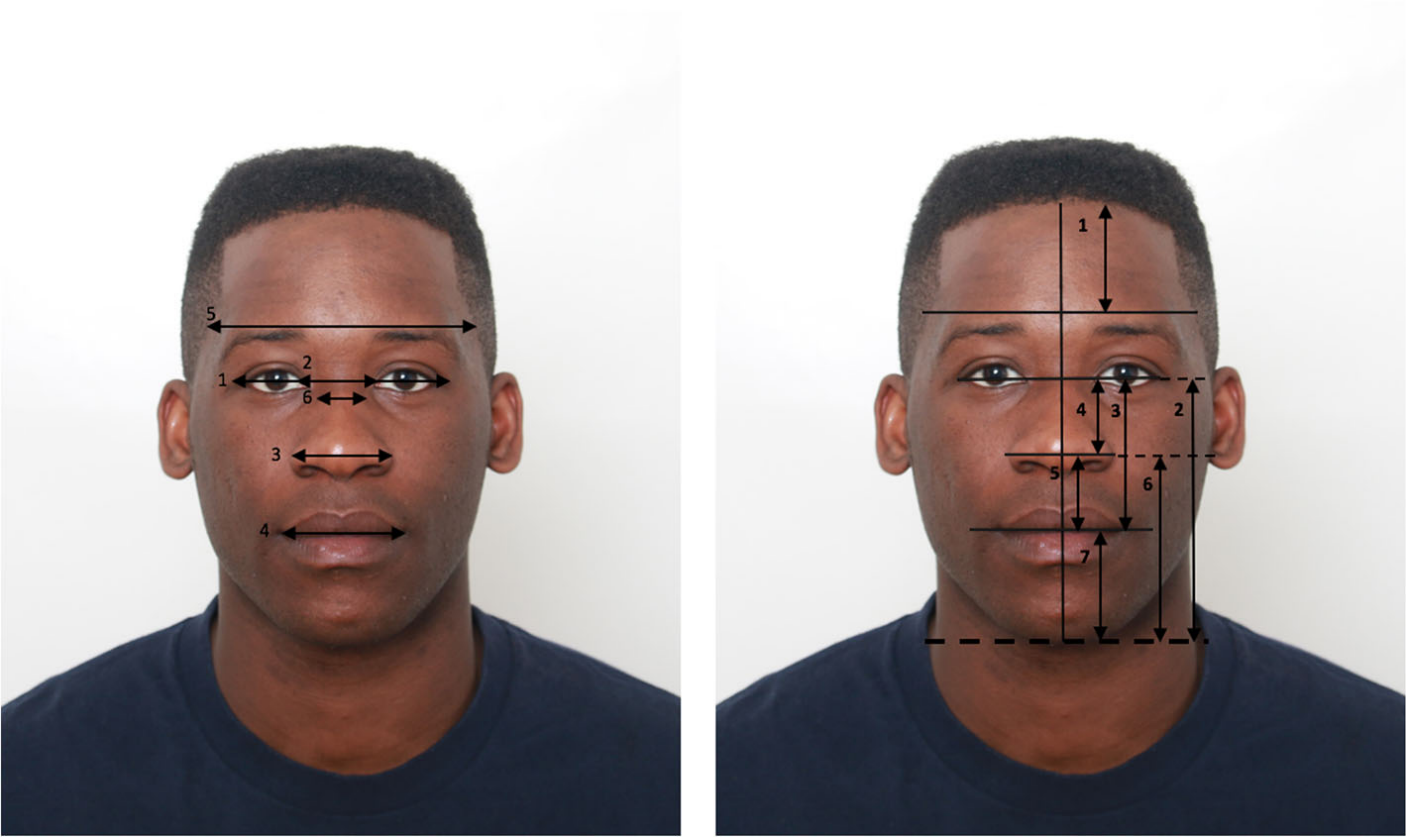
**a** Horizontal measurements (see Tables 1 and 2). **b** Vertical measurements (see Tables 1 and 3)

For each sample of the photographs, the Ricketts [4] method was used to measure the golden proportions in the vertical and horizontal facial planes (Fig. 1). Table 1 indicates the seven facial reference landmarks that were used.

**Table 1:** Definition of facial landmarks

Anthropometric landmark	Definition
Lateral canthus	The most lateral point where the superior and inferior eyelids meet
Soft tissue dacryon	The most medial point where the superior and inferior eyelids meet (i.e. medial canthus)
Soft tissue borders of temporal bone	Most lateral borders of the face in the temporal region
Lateral alae	The most lateral points on the rims of the wings of the nose
Cheilion	The point located at each lateral oral commissure, i.e. the angle of the mouth
Soft tissue menton	The most inferior midline point of the soft tissue chin
Trichion	The midline point at the junction of the hairline and forehead

The measurements were carried out using a Facial Ratio Calculator, Python®, whereby the appropriate landmarks may be inserted, and the facial measurements and ratios described in Tables 2 and 3 obtained. This was performed to reduce the chances of measurement errors from individual human measurements.

**Table 2:** Definition of horizontal facial measurements (see Fig. 1a)

Horizontal anthropometric measurements	Definition
1. Intercanthal	The horizontal measurement from the left lateral canthus to the right lateral canthus.
2. Interdacryon	The horizontal measurement between the eyes from the left dacryon to the right dacryon
3. Interalae	The horizontal measurement between the left lateral rims of the ala of the nose to the right lateral rim of the nose
4. Intercheilion	The horizontal measurements from the left cheilion to the cheilion of the mouth
5. Intertemporal	The horizontal measurement from the soft tissue lateral border of the left temple to the soft lateral tissue lateral border of the right temple measured along a line that passed through the estimated location of the supraorbital foramen.
6. Nose width (nasal dorsal width)	The horizontal measurement of the width of the nose in the region of the bony dorsum

**Table 3:** Definitions of vertical facial measurements (see Fig. 1b)

Vertical anthropometric measurements	Definition
Forehead height (vertical measurement 1)	Trichion to the line bisecting the intertemporal line
Vertical measurement 2	Intereye point to soft menton *(Intereye point: The midpoint of the intercanthal measurement)*
Vertical measurement 3	Intereye point to stomion
Vertical measurement 4	Intereye point to ala point *(Ala point: The midpoint of the interalae measurement)*
Vertical measurement 5	Ala point to stomion
Vertical measurement 6	Ala point to soft menton
Vertical measurement 7	Stomion to soft menton

Using the above measurements, different ratios were calculated in the horizontal and vertical planes, which were then compared with the golden proportion. The following ratios were calculated in the horizontal and vertical planes:

Horizontal ratios

- Intertemporal/intercanthal
- Intercanthal/intercheilion
- Interalae/interdacryon
- Interalae/nose width
- Intercheilion/interdacryon
- Intercheilion/interalae
  Vertical ratios
- Forehead height/intereye-interalae
- Forehead height/stomion-soft menton
- Ala-soft menton/stomion-soft menton
- Intereye-interalae/interalae-stomion
- Intereye-soft menton/ interalae-soft menton
- Intereye-soft menton/intereye-stomion

### Statistical analysis

SPSS Statistics for Windows, Version 21.0 (Armonk, NY:IBM Corp. Released 2012), Minitab v16 (Minitab Inc., USA) and Microsoft Excel (Microsoft Corporation, USA) were used to perform statistical tests and create graphs. It is estimated that for the study to have 95% power to detect between-group differences with a 6% margin of error, a sample of 260 responses would be needed. A total of 287 responses were gathered. The data were tested for normality and then parametric or non-parametric tests applied as appropriate.

The statistical significance in inter-gender, inter-age and inter-professional differences was calculated using the chi-squared test. Furthermore, significant statistical differences of ratios between the best graded (higher frequency of high scores) and least well-graded photographs were examined using the Mann-Whitney test. All ratios of the model photographs were then compared with the golden proportion using Wilcoxon signed rank test, and then for these ratios, an estimated confidence interval was calculated to produce a numerical range of the ideal ratio for each facial feature (using the median values of the models’ facial ratios). All graphs produced from these results were created with Microsoft Excel.

## Results

### Inter-sex, inter-age and inter-professional grading disparities

Table 4 illustrates the chi-squared analysis of grading of participants based on various demographic classifications. As shown in the table, the differences in grading of both black female and black male photographs between male and female participants were only just significant (*p* <  0.05). Regarding the age group disparities, two age groups were compared (under 45 years old and over 45 years old), where there were also found to be significant inter-age grading disparities (*p* <  0.05). Lastly, there were also statistically significant differences amongst student grading and orthodontists grading (*p* <  0.05).

**Table 4:** Differences in beauty perception of male and female models according to participants’ gender, age and profession

Demographic data compared	Female models (*p* value)	Male models (*p* value)
Gender	< 0.05*	< 0.05*
Age	< 0.05*	< 0.05*
Profession	< 0.05*	< 0.05*

*Significant differences between the groups compared

### Best graded photographs and least well-graded photograph disparities

Table 5 highlights the statistical comparison of each facial ratio between the best graded and least well-graded photographs with the aim to identify any statistical significant differences in the ratios, which may then correlate with facial beauty.

**Table 5:** Comparison of facial ratios between best graded and least well-graded male and female models

Female models			
Ratio	Best graded photograph medians	Least well-graded photograph medians	*p* value
Intertemporal/intercanthal	1.305	1.295	> 0.001
Intercanthal/intercheilion	1.938	1.913	> 0.001
Interalae/interdacryon	1.051	1.080	> 0.001
Interalae/nose width	2.092	1.975	> 0.001
Intercheilion/interdacryon	1.445	1.443	> 0.001
Intercheilion/interalae	1.421	1.344	> 0.001
Forehead height/intereye-interalae	1.453	1.667	< 0.001*
Forehead height/ stomion-soft menton	1.439	1.534	< 0.001*
Ala-soft menton/stomion-soft menton	1.649	1.717	> 0.001
Intereye-interalae/interalae-stomion	1.523	1.265	< 0.001*
Intereye-soft menton/interalae-soft menton	1.573	1.544	> 0.001
Intereye-soft menton/intereye-stomion	1.582	1.597	> 0.001
Male models			
Ratio	Best graded photograph medians	Least well-graded photograph medians	*p* value
Intertemporal/intercanthal	1.288	1.314	> 0.001
Intercanthal/intercheilion	1.824	1.753	> 0.001
Interalae/interdacryon	1.099	1.291	< 0.001*
Interalae/ nose width	1.908	2.115	< 0.001*
Intercheilion/interdacryon	1.614	1.709	< 0.001*
Intercheilion/interalae	1.450	1.337	< 0.001*
Forehead height/intereye-interalae	1.247	1.423	< 0.001*
Forehead height/stomion-soft menton	1.077	1.345	< 0.001*
Ala-soft menton/stomion-soft menton	1.640	1.708	> 0.001
Intereye-interalae/interalae-stomion	1.471	1.162	< 0.001*
Intereye-soft menton/interalae-soft menton	1.541	1.506	> 0.001
Intereye-soft menton/intereye-stomion	1.646	1.634	> 0.001

*Significant differences of ratio between the two sets

In the black female group, three out of the 12 ratios demonstrated significant difference between facial ratios when comparing the best graded and least well-graded photographs, namely the forehead height/intereye-interalae, forehead height/stomion-soft menton and intereye-interalae/interalae-stomion (*p* <  0.001). In the black male group, seven out of the 12 facial ratios demonstrated statistical significant differences, namely the forehead height/intereye-interalae, forehead height/stomion-soft menton and intereye-interalae/interalae-stomion, which were also significantly different in the female group, but also interalae/interdacryon, interalae/nose width, intercheilion/interdacryon, and intercheilion/interalae (*p* <  0.001).

### Compliance of facial ratios with the golden proportion

**Fig. 2:**
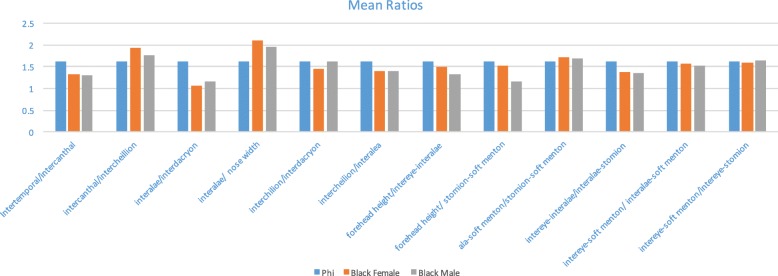
Mean ratios

Table 6 illustrates the comparison of all facial ratios of each group in comparison to the golden proportion using a Wilcoxon test. Considering that the samples selected were all professional models promoted in social media, a median value was found for each facial ratio for the two groups. Out of the 12 ratios, only one of them was similar to the golden proportion (intereye-soft menton/intereye-stomion, *p* > 0.05), whilst the rest all demonstrated statistically significant differences (Fig. 2).

**Table 6:** Comparison of facial ratios of male and female black models with the golden proportion (1.618)

Ratio	Black female model median	Female *p* value	Black male model median	Male model *p* value
Intertemporal/intercanthal	1.314	< 0.001	1.313	< 0.001*
Intercanthal/intercheilion	1.903	< 0.001	1.773	< 0.001*
Interalae/interdacryon	1.063	< 0.001	1.17	< 0.001*
Interalae/nose width	2.159	< 0.001	1.968	< 0.001*
Intercheilion/interdacryon	1.443	= 0.002	1.606	*p* = 0.695
Intercheilion/interalea	1.386	= 0.001	1.377	< 0.001*
Forehead height/intereye-interalae	1.552	= 0.173	1.303	< 0.001*
Forehead height/stomion-soft menton	1.519	= 0.05	1.176	< 0.001*
Ala-soft menton/stomion-soft menton	1.708	= 0.002	1.691	< 0.001*
Intereye-interalae/interalae-stomion	1.408	= 0.006	1.352	< 0.001*
Intereye-soft menton/interalae-soft menton	1.566	= 0.022	1.531	< 0.001*
Intereye-soft menton/intereye-stomion	1.591	= 0.151	1.639	0.323

*Significant differences of ratio between the facial ratios and the golden proportion

### Proposal of “ideal” facial ratios

**Fig. 3:**
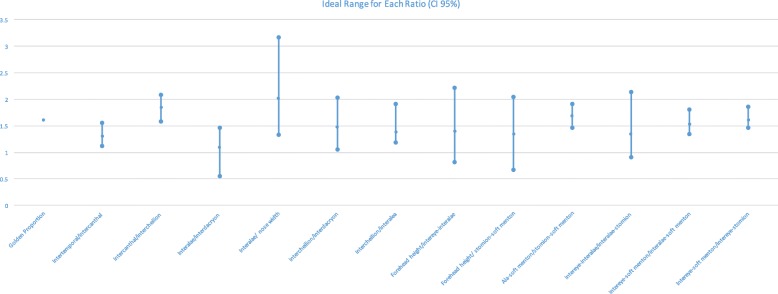
Ideal range for each ratio (95% CI)

Using the median values, in which the majority appears not to correlate with the golden proportion, it was possible to calculate the confidence interval regarding where the value of each facial ratio lies (Table 7). To do this, the median value of each ratio from the whole sample was used. This is because the median value of male and female models was closely similar (Table 6), and thus calculating different confidence interval for each gender would not necessarily provide a different result or be clinically relevant or significant. The results illustrate that there may be an “ideal” range for each facial ratio (Fig. 3).

**Table 7:** Median and range of each facial ratio of professional models

Ratio	Median	Min	Max
Intertemporal/intercanthal	1.314	1.191	1.556
Intercanthal/intercheilion	1.833	1.583	2.089
Interalae/interdacryon	1.116	0.550	1.459
Interalae/ nose width	1.992	1.357	3.174
Intercheilion/interdacryon	1.50	1.050	2.033
Intercheilion/interalea	1.389	1.188	1.909
Forehead height/intereye-interalae	1.432	0.832	2.222
Forehead height/ stomion-soft menton	1.344	0.774	2.051
Ala-soft menton/stomion-soft menton	1.701	1.535	1.909
Intereye-interalae/interalae-stomion	1.350	1.031	2.045
Intereye-soft menton/ interalae-soft menton	1.544	1.423	1.744
Intereye-soft menton/intereye-stomion	1.600	1.458	1.864

## Discussion

### Inter-sex, inter-age and inter-professional grading disparities

The concept of golden proportions being related to facial beauty is not new, and it is still proposed by some artists and sculptors as being a requirement of beauty [2]. Recently, a meta-analysis by Langlois et al. [12] published in the American Psychological Association found that facial attractiveness is a “variable” that is highly consistent between people’s judgements within and across cultures and thus established that irrespective of people’s ethnicity and cultural background, there is agreement about who is and is not attractive. This was also supported by Coetzee et al., who provided further evidence for strong cross-cultural agreement in facial aesthetics [13].

Previous investigations on facial aesthetics and the perception of beauty are extremely important; however, the subject requires modern evaluation due to the potential for changing perceptions over time. For example, female nudes from Renaissance art and sculpture would be considered potentially overweight by modern societal standards, but were appealing during their time, perhaps because a higher body mass was linked with wealth and high socioeconomic status.

#### Gender influence

We observed a difference between male and female grading of black professional models that was just significant (*p* <  0.05). This is consistent with the previous work performed by Broer et al. [14], which showed disagreement between male and female preference in lip and chin projections.

#### Profession influence

Nevertheless, discrepancy when rating facial characteristics was also just statistically significant (*p* <  0.05) between the results observed between orthodontists and students. This partly contradicts the work published by Broer et al. as in their study it was observed that male plastic surgeons in Brazil agreed with lay people regarding aesthetic perceptions [14]. It is worth mentioning that discrepancies were found between female plastic surgeons and laypeople in Brazil [14].

#### Age influence

After conducting this investigation, it was found that age is indeed a variable that appears to influence attractiveness judgement but this only just reached statistical significance (*p* <  0.05). A statistically significant disparity in the subjects’ grading behaviour was observed in the two groups compared: those younger than 45 and those who were 45 and above (*p* < 0.05).

Investigators have so far studied differences on the beholder’s age for facial characteristics preferences by comparing the preferences of infants or young children with adults, establishing that children as young as 3 months old can discriminate between unattractive and attractive faces [15, 16]. In 1992, Kolb et al. indicated similarities between the performance levels of 8 to 13-year-old children with adults with frontal lobe injury, in an expression matching task [17]. Consequently, scientists suggested that some frontal lobe regions implicated in this task may have not yet matured by this age [18]. This work has provided useful evidence for the presence of developmental factors in various aspects of face processing, since the frontal lobes are regions in the human brain which are subject to great modifications during development [18]. However, previous studies attributed frontal lobe under-development for difference in rating behaviour, whereas this difference in this study is also found in adults with fully matured and functional frontal lobe. Further research in this intriguing area of facial aesthetics and perception psychology will be significant to elucidate how the mind perceives beauty.

Various rating methods for beauty preference exist, including the traditional pair comparison method [19–21], where participants choose between two faces, as this is a lot easier than the rating method [22–24] which is more commonly used today, to measure facial attractiveness. For this study, we used the rating method as we were interested in older individuals’ perceptions. Furthermore, by using the rating method, we can further analyse our data as we will be able to identify the best rated images and the least rated images and analyse their differences in facial characteristics. In this way, we can identify proportions that are more appealing and compare them with the golden proportions. After conducting an in-depth literature review, we were not able to find information on how different adult age groups judge facial attractiveness, and for this reason, we decided to choose age groups that have not been studied before as there is the possibility of identifying important correlations in different age groups from the ones already studied.

All in all, attractiveness is a subjective measure and to date many variables have been identified that influence people’s perceptions. In our study, we discussed three main variables: gender, age and profession. It appears that all three play a role in perception of attractiveness. It is important, however, to note that these results have not taken into account that age may affect the profession, and vice versa. Further research, including all relevant factors such as age, ethnicity and cultural factors, should be performed to further explore the combined impact of all aforementioned parameters [14].

#### Disparities in best graded versus least well-graded photographs

This study examined the presence of disparities in best graded photographs in comparison with the least well-graded photographs, and we identified statistical differences in several of the facial characteristic ratios used for facial analysis. It is worth mentioning that to date, there are no studies examining the presence of such disparities in black individuals. According to our findings, black males showed statistically significant differences in seven out of the 12 ratios used for facial analysis (interalae/interdacryon, interalae/nose width, intercheilion/interdacryon, intercheilion/interalea, forehead height/intereye-interalae, forehead height/stomion-soft menton, intereye-interalae/interalae-stomion). In black females, significant differences were present in just three out of the 12 ratios (forehead height/intereye-interalae, forehead height/stomion-soft menton, intereye-interalae/interalae-stomion). Interestingly, the disparities observed in black females were present in black males, and therefore, we can conclude that there may be agreement for these ratios and perceived attractiveness, according to the people rating the photographs, regardless of the gender of the models. In other words, these three facial characteristics may partly determine attractiveness in both black males and black females.

Milutinovic et al. indicated the importance of an attractive smile as the single most important factor in an aesthetically pleasing face, for making a positive first impression [25]. The fact that females with a smaller face [25], as well as other features such as small chins [26] and noses [27] were perceived as more attractive, is also stated in the literature making it likely that attractiveness is affected by different facial features [25–27]. The literature also illustrates that feminine traits are perceived as more attractive by both males and females [27]. This study examined the presence of disparities in best graded photographs in comparison with the least well-graded photographs, and we identified statistical differences in several of the facial characteristic ratios used for facial analysis.

Further research should be undertaken to evaluate and further investigate these findings. Attractiveness is greatly appreciated by society, and this is partly because the media continuously project attractive people and therefore create the idea that attractiveness is seen as more socially acceptable [28]. As the facial proportions discussed in this article are a fundamental part of facial aesthetics, continuous research in this area is invaluable, making it possible for clinicians to advance the understanding of facial attractiveness, and exactly what are the parameters that make each face attractive. It is known that an aesthetically pleasing face is associated with greater confidence and self-esteem [29].

### Comparison of facial ratios with the golden proportion

Despite the common perception that beauty is a subjective experience [4], it is relevant for one to have clear guidelines that gauge aesthetic facial surgery to provide a consistent and accurate result for each patient. Evidence-based guidelines are not currently used in practice. Previous investigations have studied the correlation between Ricketts’ facial ratios and the golden proportion, resulting in conflicting evidence regarding the topic [8–10]. To the best of the authors’ knowledge, this is the first study to examine this correlation of facial attractiveness and the golden proportion in a black population, as all studies that have been carried out were focused on Caucasian populations.

These results of discordance of facial rations with the golden proportion agree with some relevant literature. In one study, Brazilian women were initially evaluated according to their facial attractiveness and then compared with the golden proportion, and no correlation was found between perception of beauty and the golden proportion [30]. A more recent study investigating the same topic conducted by Rossetti et al. [31] also concluded that the attractive female and male facial ratios did not correlate with the golden proportion. These results also agree with research conducted on European male and female facial proportions by Bashour [32]. Therefore, there appears to be modern research suggesting that the golden proportion cannot be applied in aesthetic facial surgery to provide consistency of results both in Caucasian and black populations.

Our results contradict findings from previous literature that illustrated that attractive faces tend to conform with the golden ratio compared to non-attractive ones. The relationship between attractive female faces and the golden proportion has also been studied by Marquardt, who created an “ideal” mask, deriving from fashion models, using the golden ratio [33]. However, this has been found to be an inaccurate and biased method to predict attractiveness [34]. As illustrated by Holland, as there are so many facial ratios one is bound to find correlations amongst some ratios with the golden ratio, making it an inherently biased method of predicting attractiveness [34]. Medici et al. [6] used facial photographs, which were modified according to the golden ratio and then ranked by judges regarding their attractiveness. It was found that the photographs with ratios that were closer to the golden proportion tended to be perceived as more attractive; however, the participant size was relatively small (12 judges involved) so the results have low statistical power. Pancherz et al. [5] also evaluated facial photographs of professional models and compared them with non-professional facial photographs, concluding that facial ratios of professional models tend to be closer to the golden proportions than non-professionals. However, as well as low statistical power, these studies demonstrate that some authors identify ratios “close to” the golden proportion as positive evidence of a link between this ratio and facial beauty.

### Proposal of ideal facial ratios

The results of this investigation demonstrate that the facial proportions of professional black models do not accurately fit the golden proportion. It is therefore relevant to identify whether any proportions are correlated with facial attractiveness, which may subsequently guide facial aesthetic surgery according to a specific guideline. Several other studies have tried to identify the ideal facial ratios to produce guidelines for future aesthetic surgery [25, 35], but these published studies only have a focus on the ideal facial shape in terms of height and width rather than identifying every individual ratio and its ideal range.

This study uniquely identifies that each facial ratio appears to be individualistic and needs to be treated separately from the others rather than attempting to find one proportion that will fit all. More specifically, the most attractive horizontal ratios found were as follows (*p* < 0.05): intertemporal/intercanthal 1.314 (1.115 to 1556), intercanthal/intercheilion 1.852 (1.583 to 2.089), interalae/interdacryon 1.099 (0.550 to 1.459), interalae/nose width 2.026 (1.33 to 3.174), intercheilion/interdacryon 1.479 (1.050 to 2.033) and intercheilion/interalaa 1.390 (1.188 to 1.909). The most attractive vertical ratios found were as follows (*p* < 0.05): forehead height/intereye-interalae 1.410 (0.811 to 2.222), forehead height/stomion-soft menton 1.346 (0.676 to 2.051), ala-soft menton/stomion-soft menton 1.699 (1.467 to 1.909), intereye-interalae/interalae-stomion 1.357 (0.912 to 2.139), intereye-soft menton/interalae-soft menton 1.540 (1.342 to 1.806) and intereye-soft menton/intereye-stomion 1.613 (1.458 to 1.864).

These findings may eventually help towards generating guidelines for aesthetic and reconstructive surgeons for patients of different ethnic backgrounds. It should be noted that most of the facial ratios evaluated in attractive male and female black subjects do not appear to conform to the golden proportion. It should also be emphasized that the results of one study are not enough to claim findings of new proportional canons—further investigation and dispassionate analysis will be required.

### Limitations

The use of a mean to generalize an ideal ratio also may be considered a limitation, as the harmony of proportions may be more important than strict, hard-ruled ratios, and should be tailored to each individual patient. Further research to consolidate the validity of the proposed ratios found in this study should be undertaken. This can be done through performing a similar methodology to a wider set of pictures or through modifying pictures according to these ratios and inviting participants to grade them before and after the modification. The data in this study was based on 2D images. Furthermore, we have assumed that no digital manipulation of the images had been undertaken by the models or their agency. 3D imaging and/or adopting standardized medical photographic techniques could influence the results.

## Conclusions

Perceptions of attractiveness between various demographic data of age, gender and profession demonstrated statistically significant differences between both groups. Comparison of best graded models with the not so well-graded models demonstrated differences in three of the 12 facial ratios in the female group, and in male models, seven out of the 12 ratios illustrated significant differences between the two groups. Only one of the 12 facial ratios was found to correlate with the golden proportion both in male and female professional models. For every facial ratio, an “ideal” range was found, illustrating the need for individualistic treatment of each facial ratio, and a requirement for improved understanding of the potential link between facial attractiveness and proportions.
